# Urban Living Environment and Myopia in Children

**DOI:** 10.1001/jamanetworkopen.2023.46999

**Published:** 2023-12-08

**Authors:** Xiaotong Li, Lihua Li, Wen Qin, Qing Cao, Xin Mu, Tiange Liu, Zhen Li, Wei Zhang

**Affiliations:** 1 Nankai University Affiliated Eye Hospital, Nankai University, Tianjin, China; 2 Tianjin Eye Hospital, Tianjin Key Lab of Ophthalmology and Visual Science, Tianjin Eye Institute, Tianjin, China; 3 Tianjin Eye Hospital Optometric Center, Tianjin, China; 4 Department of Radiology and Tianjin Key Laboratory of Functional Imaging, Tianjin Medical University General Hospital, Tianjin, China

## Abstract

**Importance:**

The global prevalence of myopia has shown a steady increase over recent decades, with urban areas seemingly experiencing a more significant impact.

**Objective:**

To assess the association between urbanization and the prevalence, incidence, progression, and severity of myopia.

**Design, Setting, and Participants:**

This cohort study included students in grades 1 to 6 in Tianjin, China, who underwent 3 vision examinations conducted over a 2-year period, from March 1, 2021, to March 31, 2023. Participants from grades 1 to 4 completed the 2-year follow-up.

**Exposures:**

Urban living environment

**Main Outcomes and Measures:**

The association of urbanization with the incidence, progression, prevalence, and severity of myopia. To quantify urbanization, an urban score was constructed using satellite data and an iterative exploratory factor analysis.

**Results:**

Of 177 894 students (51.7% male; mean [SD] age, 10.27 [1.75] years) included in the study, 137 087 students (52.3% male; mean [SD] age, 8.97 [1.21] years) were followed up for 2 years. A positive association was identified between myopia incidence and urbanization. Specifically, each 1-unit increment in the urban score was associated with an increased risk of myopia over a 1-year period (odds ratio [OR], 1.09; 95% CI, 1.01-1.15; *P* = .02) and a 2-year period (OR, 1.53; 95% CI, 1.50-1.57; *P* < .001). Conversely, each 1-unit increase in the urban score was associated with a significant decrease in myopia progression at 1 year (OR, 0.84; 95% CI, 0.82-0.86; *P* < .001) and 2 years (OR, 0.73; 95% CI, 0.70-0.75, *P* < .001). In a cross-sectional data analysis, the urban score was positively associated with myopia prevalence (OR, 1.62; 95% CI, 1.08-2.42; *P* = .02) and negatively associated with myopia severity, as indicated by spherical equivalent refraction (OR, 1.46; 95% CI, 1.07-1.99; *P* = .02).

**Conclusions and Relevance:**

This study exploring urban living environments and myopia revealed dual associations of urban living with both the incidence and the progression of myopia. The observed patterns emphasize the urgency of promptly implementing myopia control strategies in less urbanized regions, where myopia progression may be accentuated.

## Key Points

**Question:**

Is there an association between the urban living environment and the prevalence, incidence, progression, and severity of myopia?

**Findings:**

In this cohort study involving 177 894 elementary school students across diverse living environments, a significant association between higher urbanization levels and an increased risk of myopia incidence was found. Progression of myopia was slower and myopia was less severe in students in urban areas.

**Meaning:**

The findings suggest that associations between urban living and the incidence and progression of myopia exist.

## Teaser

This cohort study evaluates the association of urbanization with risk of myopia and myopia progression and with myopia severity in elementary school students.

## Introduction

Myopia has emerged as a significant global health concern over the past 3 decades, particularly in Asia, where it affects a substantial proportion of adolescents and young adults, reaching 80%-90% in urban areas of East and Southeast Asia.^1,2,3,4^ Notably, the prevalence of myopia is increasing, and the onset of myopia is occurring at younger ages. It is projected that the number of individuals with myopia was approximately 1.4 billion in 2000 and is estimated to reach 4.8 billion by 2050.^5^ While this is typically a benign condition, severe myopia is associated with elevated risk of potentially blinding conditions, such as cataracts, glaucoma, and serious retinal diseases.^6^ The increasing prevalence of myopia is largely attributed to environmental factors primarily related to changes in living environments and lifestyles.^2,3,4,5,6,7,8^ Researchers have provided evidence that residing in urban areas is associated with a higher likelihood of developing myopia.^9,10,11,12^

With the projected increase in urbanization, it is estimated that by 2050, around two-thirds of the global population will be residing in cities.^13^ This rapid urban growth brings about significant environmental changes. Urban areas are characterized by higher population densities, increased housing and building infrastructure, reduced green spaces,^14^ and more stressful social conditions.^15^ It is important to note that urban residents often have better access to health care compared with those in rural areas.^16^ While previous studies have explored the correlation between specific environmental factors associated with urban living, such as green areas, and myopia, there is a lack of understanding regarding the broader effects of the overall urban living environment on myopia.^17,18,19^ It is essential to consider the collective influence of various environmental factors within urbanization.

Satellite remote sensing techniques offer a valuable means of obtaining multidimensional information related to urbanized environments. These maps provide detailed data on various aspects of urbanization, including buildings, roads, nighttime lights, greenery, population density, and numerous other indicators.^20^ Given their ability to capture multidimensional urban living environment exposure indicators, satellite remote sensing maps present an ideal tool for extracting objective metrics for individual-level studies on urbanization.^21,22^ Building on this potential, the present study focused on exploring the association between satellite remote sensing–derived multidimensional urbanization indicators and the incidence and progression of myopia. By delving into this, the aim was to gain a clearer understanding of the association of urban living environments with myopia.

## Methods

This cohort study was conducted as part of the Investigation on Visual Habits and Eye Health Among Primary and Secondary School Students in Tianjin Project. This study underwent review and received approval from the Tianjin Eye Hospital Ethics Committee. Additionally, it was granted ethical clearance by the same committee, obviating the necessity to procure informed consent, as it was a nonmedical intervention study involving deidentified data. The study was carried out in accordance with the principles outlined in the Declaration of Helsinki.^23^ It followed the Strengthening the Reporting of Observational Studies in Epidemiology (STROBE) reporting guideline.

### Study Population

Tianjin, 1 of the 4 municipalities directly under the central government in China, spans an area of 11 966.45 km^2^ and comprises 16 districts. These districts can be categorized into central and peripheral areas, exhibiting notable disparities in living environments during the urbanization and development process (eFigure 1 in Supplement 1). Students in grades 1 to 6 from 493 schools across all districts of Tianjin were randomly selected to undergo comprehensive eye examinations in 2021. The examinations conducted for all participants involved visual acuity measurements using the Early Treatment of Diabetic Retinopathy Study (ETDRS) visual acuity chart and noncycloplegic autorefraction (ARK-700A; Nidek Corp) for both eyes. Moreover, data on personal factors, such as age, sex, grade, school socioeconomic status (with schools ranked in the top 100 in Tianjin classified as key schools and others considered normal schools), and refractive correction status (correction or uncorrection) were collected. To ensure regular vision assessments, efforts were made to conduct annual examinations for students spanning from March 1, 2021, to March 31, 2023. Initially selected students in grades 1 to 4 in 2021 who successfully completed 3 vision screenings over a 2-year period were included in longitudinal analysis. Furthermore, a questionnaire was administered to a random sample of students in grades 1 to 6 to provide additional information for the study (eTable 1 in Supplement 1). Sample size and missing value information are presented in eTable 2 in Supplement 1.

### Study Design and Data Sources

An iterative exploratory factor analysis was applied to reconstruct the urban score based on the satellite data. Initially, 12 environmental variables were derived from the data sets (eTable 3 in Supplement 1). We screened candidate environmental variables using population density as a reference, following the most recent public measure for urbanization.^24,25^ Subsequently, 4 variables were reserved and formed an urban score^26^ (eFigure 2 in Supplement 1). Detailed steps are shown in eTable 4 in Supplement 1.

**Figure 1.**
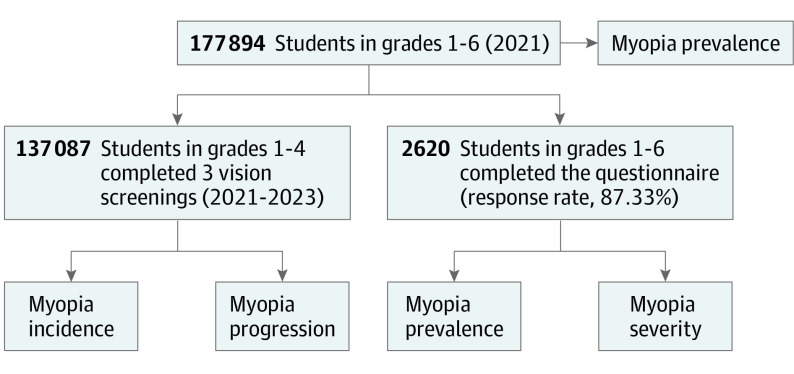
Flowchart Longitudinal and cross-sectional analyses were conducted for elementary school students in grades 1 to 4 and grades 1 to 6, respectively.

Myopia was defined as a spherical equivalent refraction (SER)≤−0.50 diopters (D) in either eye. The association between the urban living environment and myopia involved steps shown in Figure 1. The prevalence of myopia was assessed among elementary school students in grades 1 to 6, considering different urban scores. For the incidence and progression analysis using longitudinal data, adjustments were performed for age, sex, grade, baseline SER, and school socioeconomic status to assess the association between myopia incidence and the urban environment in the group without myopia at baseline. Additionally, in the group without myopia, adjustments were made for age, sex, grade, baseline SER, refractive correction status, and school socioeconomic status to examine the association between myopia progression, defined by changes in SER, and the urban environment. To further account for the influence of personal factors, information gathered through questionnaires was used. Adjustments were made for age, sex, grade, school socioeconomic status, refractive correction status, parental history of myopia, mean screen time, mean outdoor time, and academic performance to explore the association between the urban living environment and both the prevalence and the severity of myopia.

### Statistical Analysis

In this study, normal continuous variables between groups were compared using the independent samples *t* test, and nonnormal continuous variables were assessed with the Mann-Whitney U test. Additionally, the distribution of categorical variables between groups was analyzed using the χ^2^ test. Pearson correlation analyses were conducted to examine the correlations between myopia progression and factors including age, baseline SER, and urban score. Generalized mixed linear models were used to investigate the association between urban living environment indicators and myopia incidence, progression, prevalence, and severity. Two-sided tests with *P* < .05 were considered to indicate statistical significance. Data analysis and visualization were performed using R, version 4.2.0 (R Project for Statistical Computing) with the lme4 and ggplot2 packages.

## Results

**Figure 2.**
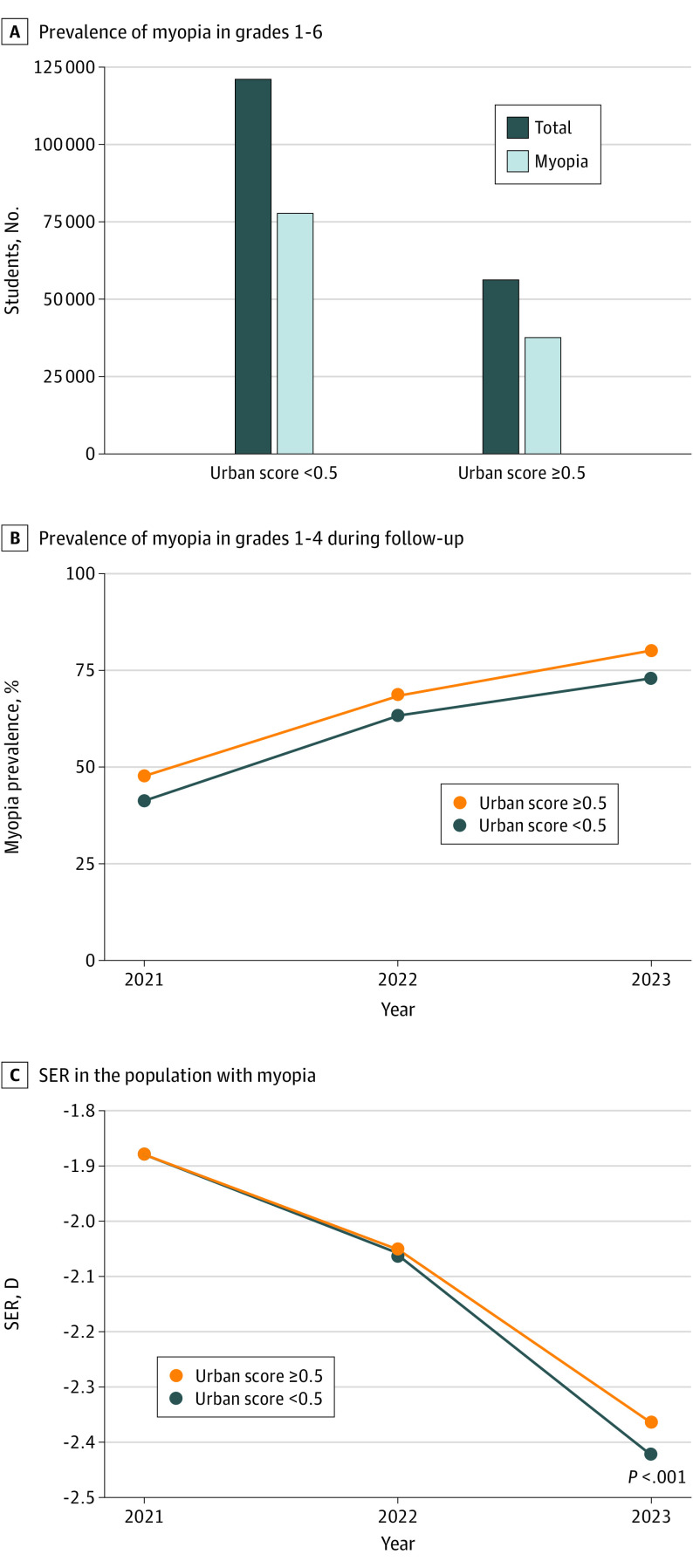
Prevalence and Severity of Myopia by High and Low Urbanization SER indicates spherical equivalent refraction.

A total of 177 894 elementary school students from grades 1 to 6 participated in this study; 48.3% were female, and 51.7% were male. The mean (SD) age of the students was 10.27 (1.75) years (eTable 5 in Supplement 1). The questionnaire was administered to a random sample of 3000 students in grades 1 to 6, resulting in a response rate of 87.3% (n = 2620). We stratified the students into 2 distinct groups based on their urban scores, which ranged from 0 to 1, with a mean (SD) of 0.42 (0.23). These groups were delineated into high urbanization (urban score ≥0.5) and low urbanization (urban score <0.5) groups. The prevalence of myopia among students living in high urbanization areas was 67.2%, while in low urbanization areas, the prevalence of myopia was slightly lower at 64.2% (Figure 2A). In the longitudinal cohort (137 087 participants from grades 1-4; 47.7% female; 52.3% male; mean [SD] age, 8.97 [1.21] years), the prevalence of myopia was consistently higher in the high urbanization group compared with the low urbanization group in 2021, 2022, and 2023. Over the 2-year period from 2021 to 2023, the prevalence of myopia increased by 32.1% in the high urbanization group, which was slightly higher than the 31.1% increase observed in the low urbanization group (Figure 2B). In the population with myopia in 2021, the mean (SD) SER was −1.878 (1.361) D in the high urbanization group and −1.879 (1.377) D in the low urbanization group, with no significant difference between the 2 groups (*P* = .48). However, after 2 years, the severity of myopia in the low urbanization group became greater than that in the high urbanization group (mean [SD] SER: −2.418 [1.674] D vs −2.362 [1.630] D; *P* < .001) (Figure 2C).

To analyze the factors associated with the incidence of myopia, individuals who did not have myopia at baseline were selected. First, a comparison was made between those who developed new-onset myopia and those who did not develop myopia during the 2-year period. The statistical results indicated that age, sex, grade, SER at baseline, school socioeconomic status, and urban score were significantly different between the groups (eTable 6 in Supplement 1). To further analyze the association between urbanization level and myopia incidence, mixed-effects logistic regression models were used while adjusting for age, sex, grade, SER at baseline, and school socioeconomic status. The results revealed a significant association between elevated urban scores and an augmented risk of myopia. For each 1-unit increment in the urban score, there was an increased risk of myopia over a 1-year period (odds ratio [OR], 1.09; 95% CI, 1.01-1.15; *P* = .02) and a 2-year period (OR, 1.53; 95% CI, 1.50-1.57; *P* < .001). Furthermore, an examination of specific indicators pertaining to urban living environments consistently bolstered these associations. Over a 2-year time frame, a 1-unit increase in the population density index was associated with a higher risk of myopia (OR, 1.56; 95% CI, 1.52-1.59; *P* < .001). Similarly, a 1-unit increase in the night light index was associated with an increased risk of myopia (OR, 1.41; 95% CI, 1.38-1.45; *P* < .001). Conversely, a 1-unit increase in the enhanced vegetation index was associated with a reduced risk of myopia (OR, 0.46; 95% CI, 0.43-0.50; *P* < .001), and a 1-unit increase in the walking time to the nearest hospital index was associated with a diminished risk of myopia (OR, 0.79; 95% CI, 0.67-0.87; *P* < .001). The consistent trend observed in these results showed an association between urban living conditions and myopia incidence (Table 1).

**Table 1.** Associations Between Multidimensional Urbanization Indicators and the Incidence and Progression of Myopia

Variable	1 y^a^		2 y^b^	
	OR (95%CI)	*P* value	OR (95%CI)	*P* value
**Urban score**				
Myopia incidence in the group without myopia at baseline^c^	1.09 (1.01-1.15)	.02	1.53 (1.50-1.57)	<.001
Myopia progression in the group with myopia at baseline^d^	0.84 (0.82-0.86)	<.001	0.73 (0.70-0.75)	<.001
**Population density**				
Myopia incidence in the group without myopia at baseline^c^	1.16 (1.09-1.22)	<.001	1.56 (1.52-1.59)	<.001
Myopia progression in the group with myopia at baseline^d^	0.88 (0.86-0.90)	<.001	0.85 (0.82-0.88)	<.001
**Night light index**				
Myopia incidence in the group without myopia at baseline^c^	1.03 (0.96-1.10)	.39	1.41 (1.38-1.45)	<.001
Myopia progression in the group with myopia at baseline^d^	0.85 (0.83-0.88)	<.001	0.76 (0.74-0.79)	<.001
**Enhanced vegetation index**				
Myopia incidence in the group without myopia at baseline^c^	0.80 (0.73-0.87)	<.001	0.46 (0.43-0.50)	<.001
Myopia progression in the group with myopia at baseline^d^	1.15 (1.11-1.18)	<.001	1.41 (1.35-1.48)	<.001
**Walking time to nearest hospital**				
Myopia incidence in the group without myopia at baseline^c^	1.16 (1.10-1.22)	<.001	0.79 (0.70-0.87)	<.001
Myopia progression in the group with myopia at baseline^d^	1.08 (1.05-1.12)	<.001	1.25 (1.21-1.30)	<.001

^a^ Of 76 937 students without myopia at baseline, 29 151 were diagnosed with myopia at 1 year. Of 60 150 students with myopia at baseline, 47 232 experienced myopia progression at 1 year.

^b^ Of 76 937 students without myopia at baseline, 43 076 were diagnosed with myopia during the 2-year follow-up. Of 60 150 students with myopia at baseline, 53 564 experienced myopia progression during the 2-year follow-up.

^c^ Mixed-effects logistic regression model adjusted for age, sex, grade, spherical equivalent refraction at baseline, and school socioeconomic status.

^d^ Generalized mixed linear model adjusted for age, sex, grade, spherical equivalent refraction at baseline, refractive correction or uncorrection, and school socioeconomic status.

Individuals who had myopia at baseline were selected to examine the association with myopia progression. Significant differences in myopia progression over the 2-year period were observed based on age, sex, grade, school socioeconomic status, and refractive correction status. Correlations between myopia progression and factors including age (*r* = −0.024; *P* < .001), baseline SER (*r* = 0.069; *P* < .001), and urban score (*r* = −0.068; *P* < .001) were observed. To account for potential confounding factors, generalized mixed linear models were adjusted for age, sex, grade, baseline SER, school socioeconomic status, and refractive correction status. The adjusted results demonstrated that each 1-unit increase in the urban score was associated with a significant decrease in myopia progression at 1 year (OR, 0.84; 95% CI, 0.82-0.86; *P* < .001) and 2 years (OR, 0.73; 95% CI, 0.70-0.75; *P* < .001). Furthermore, analyzing the specific factors related to urban living environments yielded consistent results. Living in areas with higher population density was associated with a reduction in myopia progression over 2 years per 1-unit increase in the population density index (OR, 0.85; 95% CI, 0.82-0.88; *P* < .001). Similarly, over a 2-year period of living in an urbanized living environment, a 1-unit elevation in the night light index was associated with a decrease in myopia progression (OR, 0.76; 95% CI, 0.74-0.79; *P* < .001), while an increase in the enhanced vegetation index by 1 unit was associated with an increase in myopia progression (OR, 1.41; 95% CI, 1.35-1.48; *P* < .001). Moreover, a 1-unit increase in the walking time to the nearest hospital index was associated with an increase in myopia progression (OR, 1.25; 95% CI, 1.21-1.30; *P* < .001). In summary, the findings indicated a negative association between the urban living environment and myopia progression (Table 1).

In the cross-sectional data analysis, additional factors including individual genetics and behaviors (eg, screen time, outdoor time, and academic performance) were incorporated. After adjusting for age, sex, grade, school socioeconomic status, parental history of myopia, mean screen time, mean outdoor time, and academic performance, the association between the urban living environment (as represented by urban score) and myopia prevalence was assessed. The analysis revealed that each 1-unit increase in urban score was associated with a higher myopia prevalence (OR, 1.62; 95% CI, 1.08-2.42; *P* = .02). Thus, urban living remained positively associated with myopia prevalence. Furthermore, when adjusting for age, sex, grade, school socioeconomic status, refractive correction status, parental history of myopia, mean screen time, mean outdoor time, and academic performance, it was found that individuals with myopia residing in areas with higher levels of urbanization had a lower severity of myopia as indicated by a larger SER (OR, 1.46; 95% CI, 1.07-1.99; *P* = .02). In other words, the urban living environment was negatively associated with myopia severity. A detailed overview of the results obtained from the analysis of the 4 specific factors related to the urban living environment is given in (Table 2).

**Table 2.** Associations Between Multidimensional Urbanization Indicators and Myopia Prevalence and Severity^a^

Variable	OR (95% CI)	*P* value
**Urban score**		
Prevalence^b^	1.62 (1.08-2.42)	.02
SER with myopia^c^	1.46 (1.07-1.99)	.02
**Population density**		
Prevalence^b^	1.49 (1.03-2.16)	.04
SER with myopia^c^	1.44 (1.07-1.94)	.02
**Night light index**		
Prevalence^b^	1.50 (1.06-2.13)	.02
SER with myopia	1.54 (1.17-2.02)	.002
**Enhanced vegetation index**		
Prevalence^b^	0.53 (0.36-0.80)	.002
SER with myopia^c^	0.89 (0.66-1.21)	.46
**Walking time to nearest hospital**		
Prevalence^b^	1.08 (0.71-1.65)	.73
SER with myopia^c^	0.77 (0.56-1.06)	.11

Abbreviation: SER, spherical equivalent refraction.

^a^ Of 2620 students, 1558 had myopia and were assessed for severity of myopia.

^b^ Mixed-effects logistic regression model adjusted for age, sex, grade, school socioeconomic status, parental history of myopia, mean screen time, mean outdoor time, and academic performance.

^c^ Severity was based on the SER. Generalized mixed linear model adjusted for age, sex, grade, school socioeconomic status, refractive correction or uncorrection, parental history of myopia, mean screen time, mean outdoor time, and academic performance.

## Discussion

An iterative exploratory factor analysis was conducted to reconstruct the urban score using satellite data. This involved screening and combining 4 indexes (population density, night light index, enhanced vegetation index, and walking time to nearest hospital) to construct a quantitative urbanization index. By using this urban score, the study aimed to gain a better understanding of the association between urban living environments and myopia. The findings revealed that each 1-unit increment in the urban score was associated with an increased risk of myopia over 1-year and 2-year periods. However, it was observed that urban living conditions had a mitigating association with myopia progression. Each 1-unit increase in the urban score was associated with a significant decrease in myopia progression over 1 year and 2 years. Furthermore, the results obtained from cross-sectional data analysis of individuals from grades 1 to 6 demonstrated that higher levels of urbanization were associated with a higher prevalence of myopia. Additionally, it was observed that the overall population with myopia in areas with higher levels of urbanization had a greater SER, indicating a relatively lower severity of myopia.

Previous studies have indirectly suggested that urban living contributes to an increased risk of myopia. In addition to studies that generalized to simple urban-rural divisions,^9,27,28,29^ Zhang et al^19^ found that average population density in administrative divisions was associated with myopia risk among Chinese children independent of factors such as academic activities, outdoor time, family educational level, and economic development. Similarly, studies conducted in Africa projected an increase in childhood myopia prevalence in urban settings.^30^ Additionally, the presence of green spaces within school campuses is associated with a lower prevalence of myopia at the school level and potentially with reduced risk of myopia development in individuals.^17,18^ In our study, we systematically examined various environmental variables with population density as a reference point. We included 4 specific indicators: population density, night light index, enhanced vegetation index, and walking time to nearest hospital. When analyzing these indicators individually, we observed a higher level of agreement with the results presented by the urban score. Our inclination was to place greater confidence in the notion that areas with higher population density, more intense nighttime lights, fewer green spaces, and improved medical infrastructure would be associated with a higher risk of myopia. Thus, it can be inferred that the overall urbanized living environment was associated with higher risk of myopia. However, additional research is required to ascertain whether these indicators are independently associated with myopia or whether other factors in the urbanized living environment contribute to the condition. Furthermore, our findings indicated a relatively gradual progression of myopia in highly urbanized living environments. This association may, in part, be attributed to the greater prevalence of effective myopia correction methods in urban centers. Conversely, students residing in less urbanized areas, potentially due to limited medical access, inadequate education, lack of awareness about health care, and socioeconomic challenges, may face barriers in accessing timely and appropriate myopia control measures.^31,32^ While we accounted for the impact of refractive correction status in our analysis, we only used a basic categorization of whether or not refractive correction was performed without considering the presence of undercorrection. It is important to note that undercorrection of myopia is a well-established factor that contributes to the advancement of myopia.^33,34^ Therefore, our results underscore the need for vigilant management of populations with myopia in low-urbanized areas despite the inherent risks associated with highly urbanized living environments.

### Limitations

There are certain limitations to our study that should be acknowledged. First, the optometry results used for diagnosing myopia were conducted under nonciliary muscle paralysis, which may have led to an overestimation of the prevalence of myopia among students. Second, our study relied on myopia data collected from 2021 to 2023, during which the COVID-19 pandemic impacted regular urban living environments, potentially introducing variations in the analysis of the role of urbanization compared with previous years.^35,36^ Choi et al^37^ found a correlation between the size of one’s residence and both axial length and refractive error. Nonetheless, this association could be further accentuated during specific periods of prolonged indoor activities. Third, our study merely suggests an association between urbanized living environments and the onset and progression of myopia. However, further investigation is necessary to identify the specific factors that contribute to this association.

## Conclusions

This cohort study revealed a noteworthy trend indicating a positive association between higher levels of urbanization and the likelihood of myopia occurrence. However, participants in urban areas also experienced a slower progression of myopia and lower levels of severity compared with participants in less urbanized regions. While urban living environments may contribute to an increased risk of myopia, it is imperative to stress the urgent implementation of myopia control measures in low-urbanized regions. Further research is necessary to elucidate the underlying mechanisms by which urban environments influence myopia prevalence. These findings lay a solid foundation for future interventions and urban planning strategies in addressing myopia.

## Notes

Supplement 1. **eFigure 1.** The Extent of the Population Distribution and the Images of the Urban Score in the Study **eFigure 2.** Optimal Number of Factors Estimated by Parallel Analysis **eTable 1.** Summary of the Questionnaire **eTable 2.** Missing Data Information and Sample Size **eTable 3.** Google Earth Engine Data Sets and Derived Environmental Variables **eTable 4.** Satellite Data Processing and Urban Corereconstructing **eTable 5.** Demographic Information **eTable 6.** Comparison of Factors Associated With the Incidence of Myopia

Supplement 2.
Data Sharing Statement

## References

[1] Fan Q, Verhoeven VJ, Wojciechowski R, ; Consortium for Refractive Error and Myopia. Meta-analysis of gene-environment-wide association scans accounting for education level identifies additional loci for refractive error. Nat Commun. 2016;7:11008. doi:10.1038/ncomms11008 27020472 PMC4820539

[2] Morgan IG, Ohno-Matsui K, Saw SM. Myopia. Lancet. 2012;379(9827):1739-1748. doi:10.1016/S0140-6736(12)60272-4 22559900

[3] Morgan IG, French AN, Ashby RS, . The epidemics of myopia: aetiology and prevention. Prog Retin Eye Res. 2018;62:134-149. doi:10.1016/j.preteyeres.2017.09.004 28951126

[4] Morgan IG, Rose KA. Myopia: is the nature-nurture debate finally over? Clin Exp Optom. 2019;102(1):3-17. doi:10.1111/cxo.12845 30380590

[5] Holden BA, Fricke TR, Wilson DA, . Global prevalence of myopia and high myopia and temporal trends from 2000 through 2050. Ophthalmology. 2016;123(5):1036-1042. doi:10.1016/j.ophtha.2016.01.006 26875007

[6] Saw SM, Gazzard G, Shih-Yen EC, Chua WH. Myopia and associated pathological complications. Ophthalmic Physiol Opt. 2005;25(5):381-391. doi:10.1111/j.1475-1313.2005.00298.x 16101943

[7] Rudnicka AR, Kapetanakis VV, Wathern AK, . Global variations and time trends in the prevalence of childhood myopia, a systematic review and quantitative meta-analysis: implications for aetiology and early prevention. Br J Ophthalmol. 2016;100(7):882-890. doi:10.1136/bjophthalmol-2015-307724 26802174 PMC4941141

[8] Flitcroft DI, Harb EN, Wildsoet CF. The spatial frequency content of urban and indoor environments as a potential risk factor for myopia development. Invest Ophthalmol Vis Sci. 2020;61(11):42. doi:10.1167/iovs.61.11.42 32986814 PMC7533745

[9] Ip JM, Rose KA, Morgan IG, Burlutsky G, Mitchell P. Myopia and the urban environment: findings in a sample of 12-year-old Australian school children. Invest Ophthalmol Vis Sci. 2008;49(9):3858-3863. doi:10.1167/iovs.07-1451 18469186

[10] Pan CW, Wu RK, Li J, Zhong H. Low prevalence of myopia among school children in rural China. BMC Ophthalmol. 2018;18(1):140. doi:10.1186/s12886-018-0808-0 29890943 PMC5996540

[11] Guo Y, Liu LJ, Xu L, . Outdoor activity and myopia among primary students in rural and urban regions of Beijing. Ophthalmology. 2013;120(2):277-283. doi:10.1016/j.ophtha.2012.07.086 23098368

[12] Pan CW, Shi B, Zhong H, Li J, Chen Q. The impact of parental rural-to-urban migration on children’s refractive error in rural China: a propensity score matching analysis. Ophthalmic Epidemiol. 2020;27(1):39-44. doi:10.1080/09286586.2019.1678656 31610685

[13] Heilig GK. World Urbanization Prospects: The 2011 Revision. United Nations, Department of Economic and Social Afairs, Population Division; 2012.

[14] Callaghan A, McCombe G, Harrold A, . The impact of green spaces on mental health in urban settings: a scoping review. J Ment Health. 2021;30(2):179-193. doi:10.1080/09638237.2020.1755027 32310728

[15] Badland H, Whitzman C, Lowe M, . Urban liveability: emerging lessons from Australia for exploring the potential for indicators to measure the social determinants of health. Soc Sci Med. 2014;111:64-73. doi:10.1016/j.socscimed.2014.04.003 24762261

[16] Chen L, Chen T, Lan T, Chen C, Pan J. The contributions of population distribution, healthcare resourcing, and transportation infrastructure to spatial accessibility of health care. Inquiry. 2023;60:469580221146041. 36629371 10.1177/00469580221146041 PMC9837279

[17] Zhang C, Wang C, Guo X, Xu H, Qin Z, Tao L. Effects of greenness on myopia risk and school-level myopia prevalence among high school-aged adolescents: cross-sectional study. JMIR Public Health Surveill. 2023;9:e42694. doi:10.2196/42694 36622746 PMC9871879

[18] Yang Y, Chen W, Xu A, . Spatial technology assessment of green space exposure and myopia. Ophthalmology. 2022;129(1):113-117. doi:10.1016/j.ophtha.2021.07.031 34352303

[19] Zhang M, Li L, Chen L, . Population density and refractive error among Chinese children. Invest Ophthalmol Vis Sci. 2010;51(10):4969-4976. doi:10.1167/iovs.10-5424 20445117

[20] Yuan F, Sawaya KE, Loeffelholz BC, Bauer ME. Land cover classification and change analysis of the Twin Cities (Minnesota) Metropolitan Area by multitemporal Landsat remote sensing. Remote Sens Environ. 2005;98(2):317-328. doi:10.1016/j.rse.2005.08.006

[21] Kamusoko C. Importance of remote sensing and land change modeling for urbanization studies. In: Urban Development in Asia and Africa 3-10. Springer; 2017. doi:10.1007/978-981-10-3241-7_1

[22] Mountrakis G, Im J, Ogole C. Support vector machines in remote sensing: a review. ISPRS J Photogramm Remote Sens. 2011;66(3):247-259. doi:10.1016/j.isprsjprs.2010.11.001

[23] World Medical Association. World Medical Association Declaration of Helsinki: ethical principles for medical research involving human subjects. JAMA. 2013;310(20):2191-2194. doi:10.1001/jama.2013.281053 24141714

[24] Yang XJ. China’s rapid urbanization. Science. 2013;342(6156):310. doi:10.1126/science.342.6156.310-a 24136949

[25] UNESCO. Regional Office for Education in Asia and the Pacific. Population, migration and urbanization. Bull Unesco Reg Off Educ Asia Pac. 1982;(23):289-313. 12265662

[26] Hayton JC, Allen DG, Scarpello V. Factor retention decisions in exploratory factor analysis: a tutorial on parallel analysis. Organ Res Methods. 2016;7(2):191-205. doi:10.1177/1094428104263675

[27] Wang Y, Liu L, Lu Z, . Rural-urban differences in prevalence of and risk factors for refractive errors among school children and adolescents aged 6-18 years in Dalian, China. *Front Public Health*. Published online August 29, 2022. doi: 10.3389/fpubh.2022.917781 10.3389/fpubh.2022.917781 PMC9465045 36106164

[28] Harrington SC, Stack J, Saunders K, O’Dwyer V. Refractive error and visual impairment in Ireland schoolchildren. Br J Ophthalmol. 2019;103(8):1112-1118. doi:10.1136/bjophthalmol-2018-312573 30315130 PMC6678142

[29] Chen X, Ye G, Zhong Y, . Prevalence, incidence, and risk factors for myopia among urban and rural children in southern China: protocol for a school-based cohort study. BMJ Open. 2021;11(11):e049846. doi:10.1136/bmjopen-2021-049846 34740929 PMC8573650

[30] Kobia-Acquah E, Flitcroft DI, Akowuah PK, Lingham G, Loughman J. Regional variations and temporal trends of childhood myopia prevalence in Africa: a systematic review and meta-analysis. Ophthalmic Physiol Opt. 2022;42(6):1232-1252. doi:10.1111/opo.13035 35959749 PMC9804554

[31] Nangia V, Jonas JB, Sinha A, Gupta R, Bhojwani K. Prevalence of undercorrection of refractive error in rural Central India: the Central India Eye and Medical Study. Acta Ophthalmol. 2012;90(2):e166-e167. doi:10.1111/j.1755-3768.2010.02073.x 21470380

[32] Thulasiraj RD, Nirmalan PK, Ramakrishnan R, . Blindness and vision impairment in a rural south Indian population: the Aravind Comprehensive Eye Survey. Ophthalmology. 2003;110(8):1491-1498. doi:10.1016/S0161-6420(03)00565-7 12917162

[33] Tang SM, Zhang XJ, Wang YM, . Effect of myopic undercorrection on habitual reading distance in schoolchildren: the Hong Kong Children Eye Study. Ophthalmol Ther. 2023;12(2):925-938. doi:10.1007/s40123-022-00628-2 36574139 PMC10011230

[34] Chung K, Mohidin N, O’Leary DJ. Undercorrection of myopia enhances rather than inhibits myopia progression. Vision Res. 2002;42(22):2555-2559. doi:10.1016/S0042-6989(02)00258-4 12445849

[35] Guan H, Okely AD, Aguilar-Farias N, . Promoting healthy movement behaviours among children during the COVID-19 pandemic. Lancet Child Adolesc Health. 2020;4(6):416-418. doi:10.1016/S2352-4642(20)30131-0 32458805 PMC7190292

[36] Pang JCY, Chan ELS, Lau HMC, . The impacts of physical activity on psychological and behavioral problems, and changes in physical activity, sleep and quality of life during the COVID-19 pandemic in preschoolers, children, and adolescents: a systematic review and meta-analysis. *Front Pediatr*. 2023;11:1015943. doi: 10.3389/fped.2023.1015943 10.3389/fped.2023.1015943 PMC10038232 36969271

[37] Choi KY, Yu WY, Lam CHI, . Childhood exposure to constricted living space: a possible environmental threat for myopia development. Ophthalmic Physiol Opt. 2017;37(5):568-575. doi:10.1111/opo.12397 28643407

