# Supplementary material for: Urban Living Environment and Myopia in Children
Source: JAMA Netw Open. 2023 Dec 8;6(12):e2346999. doi: 10.1001/jamanetworkopen.2023.46999 (PMC10709769; doi:10.1001/jamanetworkopen.2023.46999)
Supplement: Supplement 1. — eFigure 1. The Extent of the Population Distribution and the Images of the Urban Score in the Study eFigure 2. Optimal Number of Factors Estimated by Parallel Analysis eTable 1. Summary of the Questionnaire eTable 2. Missing Data Information and Sample Size eTable 3. Google Earth Engine Data Sets and Derived Environmental Variables eTable 4. Satellite Data Processing and Urban Corereconstructing eTable 5. Demographic Information eTable 6. Comparison of Factors Associated With the Incidence of Myopia [file jamanetwopen-e2346999-s001.pdf]

Supplemental Online Content

Li X, Li L, Qin W, et al. Urban living environment and myopia in children.
JAMA Netw Open. 2023;6(12):e2346999.
doi:10.1001/jamanetworkopen.2023.46999

eFigure 1. The extent of the population distribution and the images of the urban score in the study

eFigure 2. Optimal number of factors estimated by Parallel Analysis.

eTable 1. Summary of the questionnaire

eTable 2. Missing data information and sample size

eTable 3. Google Earth Engine datasets and derived environmental variables

eTable 4. Satellite data processing and urban corereconstructing

eTable 5. Demographic information

eTable 6. Comparison of factors associated with the incidence of myopia

This supplemental material has been provided by the authors to give readers additional information about their work.

© 2023 Li X et al. *JAMA Network Open.*

eFigure 1.The extent of the population distribution and the images of the urban score in the study.

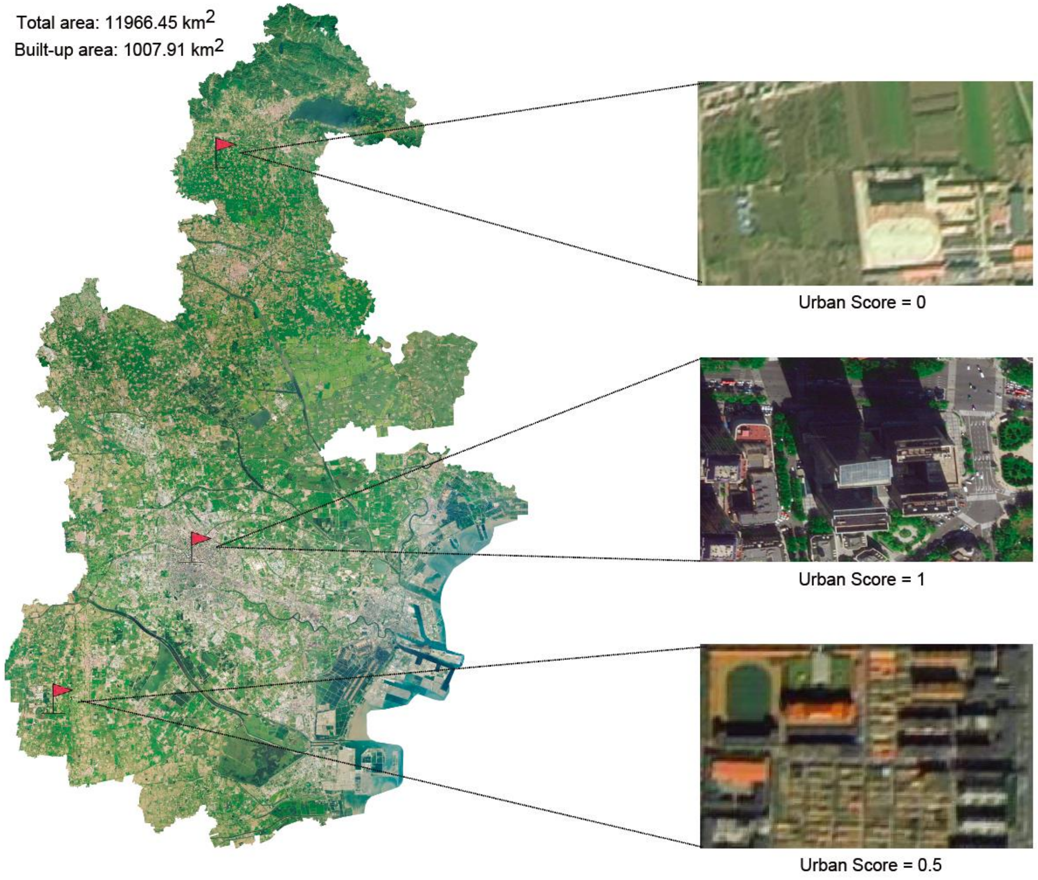

Note: The urban scores, derived from school locations, ranged from 0 to 1. A higher score indicated a more urbanized exposure, with 0 representing the lowest level of urbanization and 1 representing the highest. The mean (SD) urban score was 0.42 (0.23).

© 2023 Li X et al. *JAMA Network Open.*

eFigure 2. Optimal number of factors estimated by Parallel Analysis.

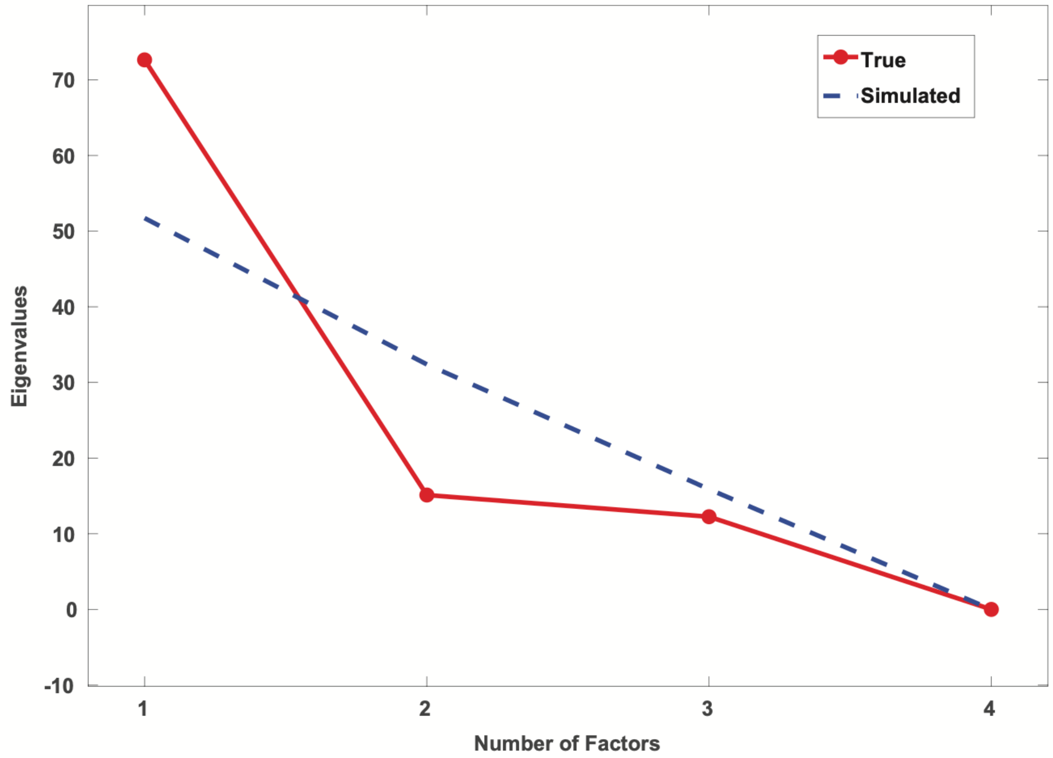

Note: 1. X axis represents the estimated number of factors, Y axis represents the eigenvalues.

2. Solid red line represents the true eigenvalue distributions, and dashed blue line represents the null eigenvalue distributions estimated by 1000 randomization. The best number of factors is determined by the max factor number whos true eigenvalue is higher than the null randomization.

3. Four variables (GPWpop, AHCw_mean, L7_EVI, and NL) were reserved and formed one optimal composite factor (KMO=0.652, P value of Bexartlett chi-square test<0.001, RMSR=0.037, goodness of fit index [GFI]=0.996, and Bayes information criterion [BIC] =103.776).

© 2023 Li X et al. *JAMA Network Open.*

eTable 1. Summary of the questionnaire

Questions	Sample	SER*(diopters), median (IQR)	P-value†
Q1:How much time have you spent outdoors during the day in the past 7 days? ____________ (mins/per day); The corresponding option is ____________	2620	-0.75(-1.9,-0.1)	
A. < 1 hour	1270	-0.63(-1.9,-0.1)	0.134
C. 1≤ time< 2 hours	462	-0.69(-1.9,-0.1)	
D. 2≤ time< 3 hours	234	-0.88(-2.5,-0.3)	
E. ≥ 3 hours	654	-0.63(-1.9,-0.1)	
Q2: How much screen time have you spent during the day in the past 7 days? (Including computer, iPad, and TV) ______________(mins/per day); The corresponding option is _______________	2620	-0.75(-1.9,-0.1)	
A. < 1 hour	225	-0.63(-1.8,-0.1)	0.172
B. 1≤ time< 2 hour	777	-0.75(-2.3,-0.1)	
C. 2≤ time< 3 hour	1074	-0.63(-1.9,-0.1)	
D. ≥ 3 hours	544	-0.63(-1.8,-0.1)	
Q3: What has been your academic ranking in school this semester? _____________ (Rank/Total); The corresponding option is ____________	2620	-0.75(-1.9,-0.1)	
A. in the top 1/3	830	-0.75(-1.9,-0.1)	0.002
B. in the middle	1580	-0.63(-1.9,-0.1)	
C. in the bottom 1/3	210	-1.00(-2.9,-0.3)	
Q4: Are either of your parents affected by nearsightedness exceeding 6.0 diopters(-6.00D)? The corresponding option is __________. If so, their refractive error is _______D	2620	-0.75(-1.9,-0.1)	
A. Yes	1303	-0.88(-2.3,-0.1)	<0.001
B. No	1317	-0.63(-1.8,-0.1)	

*SER: spherical equivalent refraction;

†The P-value represents the assessment of the variability of spherical equivalent refraction among the different options using a nonparametric test.

© 2023 Li X et al. *JAMA Network Open.*

eTable 2. Missing data information and sample size

**Elementary school students in years 1-6, 2021**	**Total sample (n=179540, missing data cases=1646)**	**Population included (n=177,894)**	**P-value***
Grade, median (IQR)	4(2,5)	4(2,5)	1
Sex, (Female%)	48.20%	48.30%	1
With or without glasses （glasses%)	17.50%	17.50%	1
Longitudinal data	(n=139,167, missing data cases=2080)	n=137,087	
Grade, mean (SD)	3(1,2)	3(1,2)	0.95
Sex, (Female%)	47.50%	47.70%	0.43
With or without glasses （glasses%)	9.70%	9.70%	0.91

*The P-value using a nonparametric test.

Note: 1. Baseline sample size calculation: The total populationof approximately 900,000 elementary school students in Tianjin. Utilizing a sampling rate of 20% (in accordance with the guidelines outlined in the Chinese Children's Vision Screening Exercise, which specify the required percentage of sampling), this methodological approach resulted in an estimated total sample size of 18,000.

2. Missing values were mainly for age, spherical equivalent refraction.

3.Missing values were addressed by employing the method of removing cases with missing data

© 2023 Li X et al. *JAMA Network Open.*

eTable 3. Google Earth Engine datasets and derived environmental variables

Variable Abbreviations	Variable Description
**Accessibility to HealthCare 2019 (AHC2019)**	
AHCw_mean	Travel time to the nearest hospital or clinic using non-motorized transport
AHC_mean	Travel time to the nearest hospital or clinic
**GPWv411 UN WPP-Adjusted Population Density (GPWv411-PD)**	
GPWpop	Estimates of population density based on counts consistent with national censuses and population registers with respect to relative spatial distribution, but adjusted to match United Nations country total
**USGS Landsat 7 Surface Reflectance Tier 1 (LandSat7-SR)**	
L7_EVI	Enhanced Vegetation Index
L7_NDVI	Normalized Difference Vegetation Index
L7_NDWI	Normalized Difference Water Index
L7_LSWI	Land Surface Water Index
L7_NDBI	Normalized Difference Built-up Index
L7_SAVI	Soil Adjusted Vegetation Index
L7_OSAVI	Optimized Soil Adjusted Vegetation Index
L7_IBI	Index-Based Built-up Index
**VIIRS Stray Light Corrected Nighttime Day Night Band Composites Version 1 (VNLv1)**	
NL	Average Day Night Band (DNB) radiance values

© 2023 Li X et al. *JAMA Network Open.*

eTable 4. Satellite data processing and urban corereconstructing

Variables	Procedures
Satellite data	1. Four candidate satellite databases related to urbanicity were selected from the Google Earth Engine (GEE)., including: GPWv411 UN WPP-Adjusted Population Density (GPWv411-PD), USGS Landsat 7 Surface Reflectance Tier 1 (LandSat7-SR), Accessibility to Healthcare 2019 (AH2019), and VIIRS Stray Light Corrected Nighttime Day Night Band Composites Version 1 (VNLv1);<br>2. Twelve environmental variables were derived from the four datasets for each school location ranging within 1 km2;<br>3. Population density (GPWpop) was used as a reference to screen candidate environmental variables for urbanization based on the following two criteria:<br>1) whose pearson correlation coefficient with GPWpop >= 0.3;<br>2) non-collinearity with other variables (r < 0.95);<br>4. Six variables were kept after this screening strategy (GPWpop, AHCw_mean, AHC_mean, L7_EVI, L7_LSWI, and NL).
**Urban corereconstructing**	1. The validity of EFA was evaluated , if the Kaiser-Meyer-Olkin (KMO) index >0.5 or P value of Bartlett's test of sphericity < 0.05, the variable with the minimum average cross-correlation coefficient were removed;<br>2. The optimal number of factors was estimated based on parallel analysis using 1000 randomization to generate the null eigenvalue distributions;<br>3. An EFA was estimated using oblique Procrustes rotation (Promax). If the root mean square of residuals (RMSR) was higher than 0.05, then the variable with the maximum specific variance was removed;<br>4. The upper three steps repeated until no variable was removed;<br>5. Four variables (GPWpop, AHCw_mean, L7_EVI, and NL) were reserved and formed one optimal composite factor;<br>6. The four environmental variables of each child at each age were extracted and z-scored across children. Then the individual composite urban score (US) was calculated based on equation：<br>$$US_{ia} = \frac{\sum_{v=1}^{N} \mathrm{E}_{iav} \times \mathrm{w}_v}{N}$$<br>In which E_{iav} represents the z-score of environmental variable for v subject i at age a , w_v represents the EFA loading of environmental variable v , and N represents the number of involved variables (N=4 in this study).

© 2023 Li X et al. *JAMA Network Open.*

eTable 5. Demographic information

**Elementary school students in grades 1 to 6（2021）**			
Variables	N	Mean	SD
Age	177894	10.27	1.75
Grade	177894	3.81	1.67
Sex (Male/ Female)	92030(51.7%)/85864(48.3%)		
Socioeconomic status (Key/ Normal)	24572(13.8%)/153322(86.2%)		
Refractive Correction Status (Correction/ Myopia)	29719(25.6%)/115994		
Spherical Equivalent Refraction (Diopters)	177894	-1.41	1.66
**Students in grades 1-4 at baseline of the longitudinal study**			
Variables	N	Mean	SD
Age	137087	8.97	1.21
Grade	137087	2.52	1.11
Sex (Male/ Female)	71676(52.3%)/65411(47.7%)		
Socioeconomic status (Key/ Normal)	18467(13.4%)/118620 (86.6%)		
Refractive Correction Status (Correction/ Myopia)	13428(9.7%)/60150		
Spherical Equivalent Refraction	137087	-0.72	1.45
**Students in grades 1 to 6 participating in a questionnaire survey**			
Variables	N	Mean	SD
Age	2620	10.23	1.89
Grade	2620	3.72	1.7
Sex (Male/ Female)	1253(47.8%)/1367(52.2%)		
Socioeconomic status (Key/ Normal)	309(11.8%)/2311(88.2%)		
Refractive Correction Status (Correction/ Myopia)	372(23.8%)/1558		
Spherical Equivalent Refraction	2620	-1.19	1.69

© 2023 Li X et al. *JAMA Network Open.*

eTable 6. Comparison of factors associated with the incidence of myopia

Variables	Non-onset	New-onset	P-value†
Age (years), median (IQR)	8.968(7.979,9.967)	8.980(7.989,9.978)	<0.001
Grade (Grade 1[%], 2[%], 3[%], 4[%])	34.32%,28.01%, 21.85%,15.82%	30.07%,28.09% 24.29%,17.55%	<0.001
Sex, (Female%)	34.30%	48.40%	<0.001
Socioeconomic status (Key%)	10.30%	13.90%	<0.001
SER* at baseline (Diopters)	0.250(0.000,0.075)	-0.13(-0.25,0.130)	<0.001
Urban score	0.365(0.236,0.500)	0.386(0.248,0.530)	<0.001

*SER: spherical equivalent refraction;

†The P-value represents the assessment of the variability of spherical equivalent refraction among the different options using a nonparametric test.

© 2023 Li X et al. *JAMA Network Open.*
