# Supplementary material for: Urban Living Environment and Myopia in Children
Source: JAMA Netw Open. 2023 Dec 8;6(12):e2346999. doi: 10.1001/jamanetworkopen.2023.46999 (PMC10709769; doi:10.1001/jamanetworkopen.2023.46999)
Supplement: Supplement 2. — Data Sharing Statement [file jamanetwopen-e2346999-s002.pdf]

# Data Sharing Statement

Li. Urban Living Environment and Myopia in Children. *JAMA Netw Open*. Published December 08, 2023. doi:10.1001/jamanetworkopen.2023.46999

**Data**
**Data available:** No
